# 24-Month clinical evaluation of cervical restorations bonded using radio-opaque universal adhesive compared to conventional universal adhesive in carious cervical lesions: A randomized clinical trial

**DOI:** 10.1038/s41598-025-88201-2

**Published:** 2025-02-14

**Authors:** Basma Dawoud, Eman Abou-Auf, Omar Shaalan

**Affiliations:** Conservative Dentistry Department, Faculty of Dentistry, Cairo University, Al Saraya Str. 11, Manial, Cairo, Egypt

**Keywords:** Cervical, Class V, Clinical performance, Universal adhesive, USPHS criteria, Dental materials, Restorative dentistry, Dentistry

## Abstract

The aim of the current study was to evaluate the clinical performance of the novel radio-opaque universal adhesive “Scotchbond™ Universal Adhesive Plus” compared to conventional universal adhesive “Single Bond Universal” over 24 months in cervical carious lesions. Fifty participants with cervical carious lesions were randomly allocated into two groups (*n* = 25); either Scotchbond™ Universal Plus Adhesive (intervention) or Single Bond™ Universal Adhesive (control). Restorations were assessed at baseline, 12 and 24 months using the modified USPHS criteria. Data analysis was conducted using MedCalc software, version 22 for Windows. Intergroup comparisons at each follow-up were performed using the Chi-Square test (*p* ≤ 0.05). Intragroup comparisons within each intervention were conducted using Cochran’s Q test (*p* ≤ 0.016). After 24 months, all restorations in Scotchbond™ Universal Plus scored alpha, while in Single Bond™ Universal group, three restorations scored bravo after 24 months in marginal adaptation and discoloration. There was no statistically significant difference between both adhesives (*p* > 0.05) at all follow-up periods. Intragroup comparison within both adhesives has shown no statistically significant change across follow-up periods regarding all tested outcomes (*p* > 0.016) except for marginal adaptation within Single Bond Universal, where there was statistically significant difference (*p* = 0.005). Both adhesives exhibited satisfactory clinical performance in cervical restorations after 24-months. The present study emphasizes the clinical significance of using a new radio-opaque universal adhesive for restoring carious cervical lesions, providing radio-opacity, low viscosity, excellent handling, eliminating misinterpretation of MDP-based adhesive layer and generating reliable bonding performance to support long-term success in restorative dentistry.

## Introduction

Over recent decades, carious cervical lesions have been treated with various materials, such as glass-ionomer cements, resin-modified glass-ionomer cements, giomers and resin composites^1^. Resin composites are frequently used for class V lesions due to their aesthetic potentials and capacity of bonding to both enamel and dentin^2^. Despite developments in restorative materials and techniques, restoring cervical lesions remains challenging. The clinical success of cervical composite restorations can be affected by several factors, including resin composite type, adhesive technique, tooth type, and the operator’s experience and skills^3^. Cervical cavities present a unique challenge due to their non-retentive form and margins that terminate on dentin or cementum, which are less ideal for bonding and located close to the gingival margin^4^.

One of the primary difficulties with cervical lesions is achieving a complete seal of the cavity, which can lead to microleakage. This microleakage may cause issues such as marginal discoloration and recurrent caries^5^, the marginal failure of resin-based composites is often related to the quality of adhesion to tooth tissues^6^. Adhesive systems play a crucial role in the success of resin composite restorations, prompting the development of various systems aimed at enhancing the bonding performance^7^.

The introduction of universal adhesives, the latest generation of adhesive systems, has simplified bonding procedures. Universal adhesives are adaptable systems that can be used in self-etch, selective-etch, or etch-and-rinse bonding strategies^8^, allowing for reduced chair time and lower technique sensitivity, while maintaining reliable long-term bonding durability^9^. Moreover, the inclusion of functional monomers, such as 10-methacryloyloxy-decyl-dihydrogen-phosphate (10-MDP), enhances bonding capabilities to various substrates, including resin composites, glass ceramics, zirconia, and metal alloys^10^.

Current research has largely focused on assessing the effectiveness and longevity of different materials to help practitioners select the most suitable options for clinical use. However, evidence remains inconclusive in endorsing a single material as the standard for restoring cervical lesions^11^. Consequently, there is ongoing work to enhance the physical and adhesive properties of adhesive systems, drivenby the dental market’s response to increased demand for adhesive advancements^12^.

A recent development in this field is the modified radio-opaque universal adhesive, Scotchbond™ Universal Plus Adhesive. This new formulation of universal adhesive has been modified by incorporating a new crosslinking resin, containing brominated dimethacrylate monomer providing dentin like radio-opacity, eliminating misinterpretation of MDP-based adhesive layer. Moreover, unlike other radio-opaque adhesive, it remains homogenous ensuring low viscosity and favourable handling while maintaining good mechanical properties. These benefit are accompanied with versatility, reduced technique sensitivity and enhanced durability present in the conventional universal adhesive^13^. As with any new material, evidence-based data for this product remains limited, highlighting the need for clinical trials to establish consistent findings regarding its bonding performance in the oral environment after modifications in the chemical formula of the previous generation of universal adhesive. Class V clinical trials are considered the gold standard for assessing adhesive performance due to their unique ability to assess bonding effectiveness without macro-mechanical retention^14^.

The current clinical trial aimed to evaluate the performance of a universal adhesive containing a novel radiopaque crosslinking resin in comparison to a conventional universal adhesive over 24 months for restoring cervical carious lesions. The null hypothesis proposed was that there would be no difference in clinical performance between the radiopaque universal adhesive and the conventional universal adhesive in cervical restorations.

## Materials and methods

### Trial registration and study setting

The protocol for the present study was submitted to the clinical trials registry NCT05509127 (19-08-2022) and received ethical approval from the Research Ethics Committee at the Faculty of Dentistry, Cairo University (18-11-22). The study was conducted within the Department of Conservative Dentistry.

#### Trial design

This study was a randomized clinical trial with a parallel, two-arm design, using a 1:1 allocation ratio and a superiority framework and reported according to the CONSORT guidelines^15^.

#### Recruitment

Participants were recruited from the diagnostic center using convenient consecutive sampling based on the eligibility criteria between May 2022 and July 2022. Prior to enrollment, all candidates were informed about the study procedures and possible harms. Informed consent was obtained by having them sign an Arabic version of the consent form. Intervention was implemented between August 2022 and November 2022.

#### Sample size calculation

The sample size was calculated based on a previous study^16^, in which success rate of resin composite cervical restorations using universal adhesive in total etch mode was 100% after 24 months. A two-tailed Z test was conducted to determine the difference between two independent proportions, with a 5% significance level (alpha) and 80% power. The minimum sample size required was 22 per group to detect a 30% difference. To account for potential dropouts, the sample size was increased by 15%, resulting in 25 teeth per group. Sample size calculation was performed using G*Power version 3.1.9.2 for Windows.

### Eligibility criteria

#### Participants

Inclusion criteria of participants in the study was to have carious cervical lesions in maxillary premolar teeth, age range between 20 and 40 years old, and exhibit only mild to moderate plaque accumulation according to Silness and Löe Plaque Index including score 0, 1 and 2. Exclusion criteria was cervical carious lesions in anterior teeth, molars, or mandibular teeth; non carious cervical lesions; any systemic conditions; allergies to resin; non-compliance; potential pregnancy; poor oral hygiene; heavy smoking; xerostomia; parafunctional habits or bruxism; and temporomandibular joint disorders.

#### Teeth

Teeth eligible for inclusion had small to moderate carious cervical lesions using visual-tactile examination (ICDAS scores 3 and 4)^17^, vital maxillary premolars, and favorable occlusion with normal occlusal contact. Exclusion criteria of teeth included deep caries close to the pulp (less than 1 mm), irreversible pulpitis or pulp necrosis, dentin hypersensitivity, the likelihood of future prosthetic restoration on the teeth, and severe periodontal conditions.

#### Sequence generation and allocation concealment

Simple randomization method was used to generate numbers from 1 to 50 via the website (https://www.random.org/sequences) using random sequence generator and divided into two columns, designating either the intervention or control group. Allocation was concealed from the operating dentist, who selected a number from a sealed, opaque envelope. The current study was double blinded to both participants and outcome assessors who did not participate in any of the procedural steps in the present study. Implementation was done by a resident who was not involved in any of procedural steps or outcome assessment in the current trial.

#### Intervention

##### Tooth preparation

Teeth to be prepared were anesthetized using buccal infiltration with a short needle and local anaesthetic solution (Articaine HCL 4% 1:100.000, Art Pharma Dent Pharmaceuticals, Giza, Egypt). Optimum shade of the restoration was selected before rubber dam isolation using direct mock-up technique using cured resin composite buttons, which was compared to the tooth and the closest shade to the tooth was determined^18^.The teeth were then isolated with a rubber dam using the quadrant isolation technique, with a subgingival clamp employed for gingival retraction on the offending tooth. Class V cavity was prepared with a #330 or #245 bur using a high-speed contra-angled handpiece with oil-free air/water coolant. Mesio-distal width was limited before the labio-proximal line angles to avoid extending to the proximal surface, and occluso-gingival length was restricted to the cervical one-third. Carious tissue was eliminated till reaching firm affected dentin using low-speed large round carbide bur for hard carious dentin or sharp excavator for soft carious dentin, direction of excavation was from the periphery to the centre to avoid pulp exposure. The occlusal cavity margin was bevelled using a yellow-coded tapered finishing stone (TR-12). Each bur or diamond point was discarded after a maximum of five teeth^19^.

##### Adhesive procedures

Enamel margins were etched for 15 s and dentin for 10 s with 3M™ Scotchbond™ Universal Etchant Gel. The surface was then rinsed for 15 s and dried with oil-free compressed air for an additional 15 s following the manufacturers’ recommendations. Afterwards Scotchbond™ Universal Plus Adhesive and Single Bond Universal Adhesive were applied according to the manufacturer’s instructions. Both adhesives were applied with a micro-brush, then agitated with the brush for 20 s, this was followed by gentle air-thinned for 5 s, and then light-curing with an LED curing unit (I-LED, Woodpecker, Guangxi, China) for 20 s.

##### Restoration

**Fig. 1:**
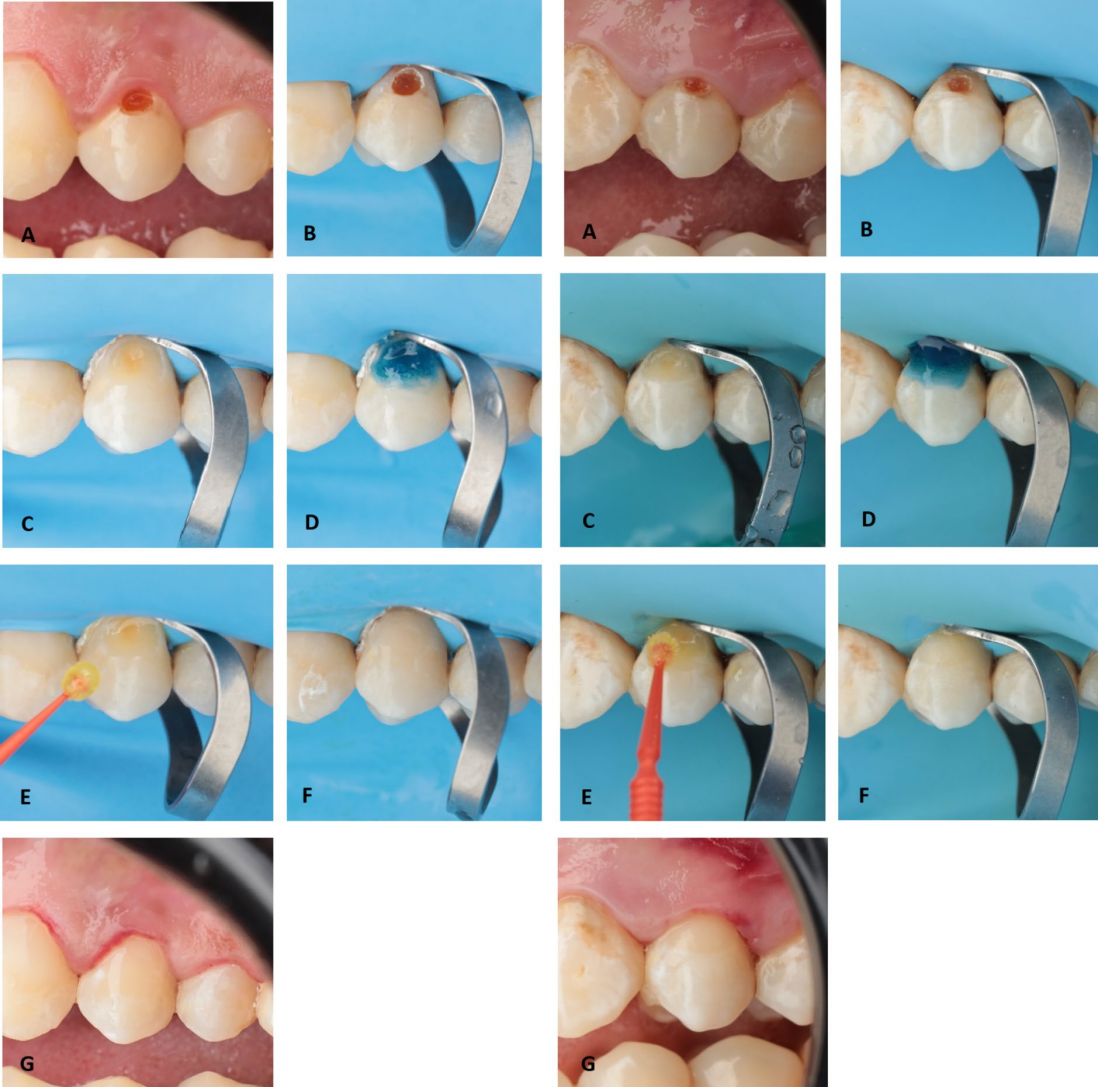
Left side: Scotchbond Universal Plus group; Right side: Single Bond Universal group. (**A**) Pre-operative; (**B**) Isolation; (**C**) Cavity preparation; (**D**) Etching; (**E**) Adhesive application; (**F**) Restoration; (**G**) Immediate post-operative after finishing and polishing.

To standardize the restoration, a nano-filled resin composite (3M™ Filtek Z350 XT) was applied in both groups. Resin composite was placed in a maximum of 2 mm increments using dentin and enamel shades, dentin shade increment was cured for 40 s and enamel shade increment was cured for 20 s with an LED curing unit. Excess composite was removed using #12 blade, followed by a fine diamond bur (TR-12). Finishing and polishing were done with TOR VM discs on a low-speed handpiece with air/water coolant, following this sequence: coarse (70–90 μm), medium (40 μm), fine (24 μm), and super-fine (8 μm) aluminum oxide discs. Polishing was carried out using pre-impregnated rubber cups with intermittent water spray. (OneGloss PS, Shofu, California, USA). Table 1 provides the names, descriptions, compositions, lot numbers, and manufacturers of the materials used. Figure 1 shows restorative procedures in both groups.

**Table 1:** Materials’ description, composition, lot number and manufacturer.

Material	Composition	Lot number	Manufacturer
Scotchbond™ universal etchant (etching gel)	32% phosphoric acid	8019534	3M Deutschland GmbH, Neuss, Germany.
Scotchbond™ universal plus adhesive (one-step universal adhesive)	10-MDP phosphate monomer, Vitrebond copolymer, HEMA, brominated dimethacrylate resin, dual-cure accelerator, camphorquinone, optimized silane, ethanol, and water.	7910510	3M Deutschland GmbH, Neuss, Germany
Single bond universal adhesive (one-step universal adhesive)	10-MDP phosphate monomer, Vitrebond, copolymer, HEMA, Bis-GMA, dimethacrylate resin, camphorquinone, silane, ethanol, and water	20524 A	3 M Deutschland GmbH, Neuss, Germany
Filtek Z350 XT (nano-filled resin composite)	Bis-GMA, UDMA, TEGDMA, Bis-EMA, non-agglomerated/ non-aggregated 20 nm silica filler, non-agglomerated/non-aggregated 4 to 11 nm zirconia filler, and aggregated zirconia/ silica cluster filler.	NF26118	3M ESPE, St. Paul, MN, USA

*MDP* methacryloxydecyl dihydrogen phosphate, *HEMA* hydroxyethyl methacrylate, *Bis-GMA* bisphenol A glycidyl methacrylate, *UDMA* urethane dimethacrylate, *TEGDMA* tri-ethylene glycol dimethacrylate, *Bis-EMA* bisphenol A ethoxylated diglycidyl methacrylate.

#### Outcome assessment

The restorations were evaluated using modified USPHS criteria by two trained, calibrated, and blinded assessors with PhD degree and 15 years of experience in restorative dentistry, they are not involved in any procedural steps. Restorations were assessed at baseline, 12 and 24 months according to the outcome chart supplied (Table 2). When assessors disagreed on a score, they discussed to reach a consensus. After training and calibration of assessors, interobserver agreement has shown Kappa coefficient of 0.92, which is nearly perfect agreement.

**Table 2:** Modified USPHS criteria.

Outcome	Score	Characteristics
Retention	A	No loss of restorative material
	C	Missing restoration
Secondary caries	A	Absence of caries
	C	Presence of caries
Post-operative sensitivity	A	Absence of post-operative sensitivity
	C	Presence of post-operative sensitivity
Marginal adaptation	A	Margins are closely adapted, no detectable margin
	B	Clinically acceptable detectable marginal discrepancy
	C	Clinically un-acceptable marginal crevice
Marginal discoloration	A	Absence of discoloration at the tooth-restoration margin
	B	Presence of non-penetrating marginal discoloration than can be polished away
	C	Presence of marginal discoloration penetrating the tooth-restoration margin in pulpal direction

### Statistical analysis

Data analysis was conducted using MedCalc software, version 22 for Windows (MedCalc Software Ltd, Ostend, Belgium). Categorical data were presented as frequencies and percentages. Intergroup comparisons between interventions at each follow-up were performed using the Chi-Square test with a significance level of (*p* ≤ 0.05). Intragroup comparisons within each intervention between follow-up periods were conducted using Cochran’s Q test, with the significance level adjusted to (*p* ≤ 0.016) after Bonferroni correction. Relative risk (RR) was calculated to assess clinical significance. The survival rate was analysed using the Kaplan-Meier method and the Log-rank test. The confidence interval was set at 95%, with 80% power, and all tests were two-tailed.

## Results

### Demographic data

**Fig. 2:**
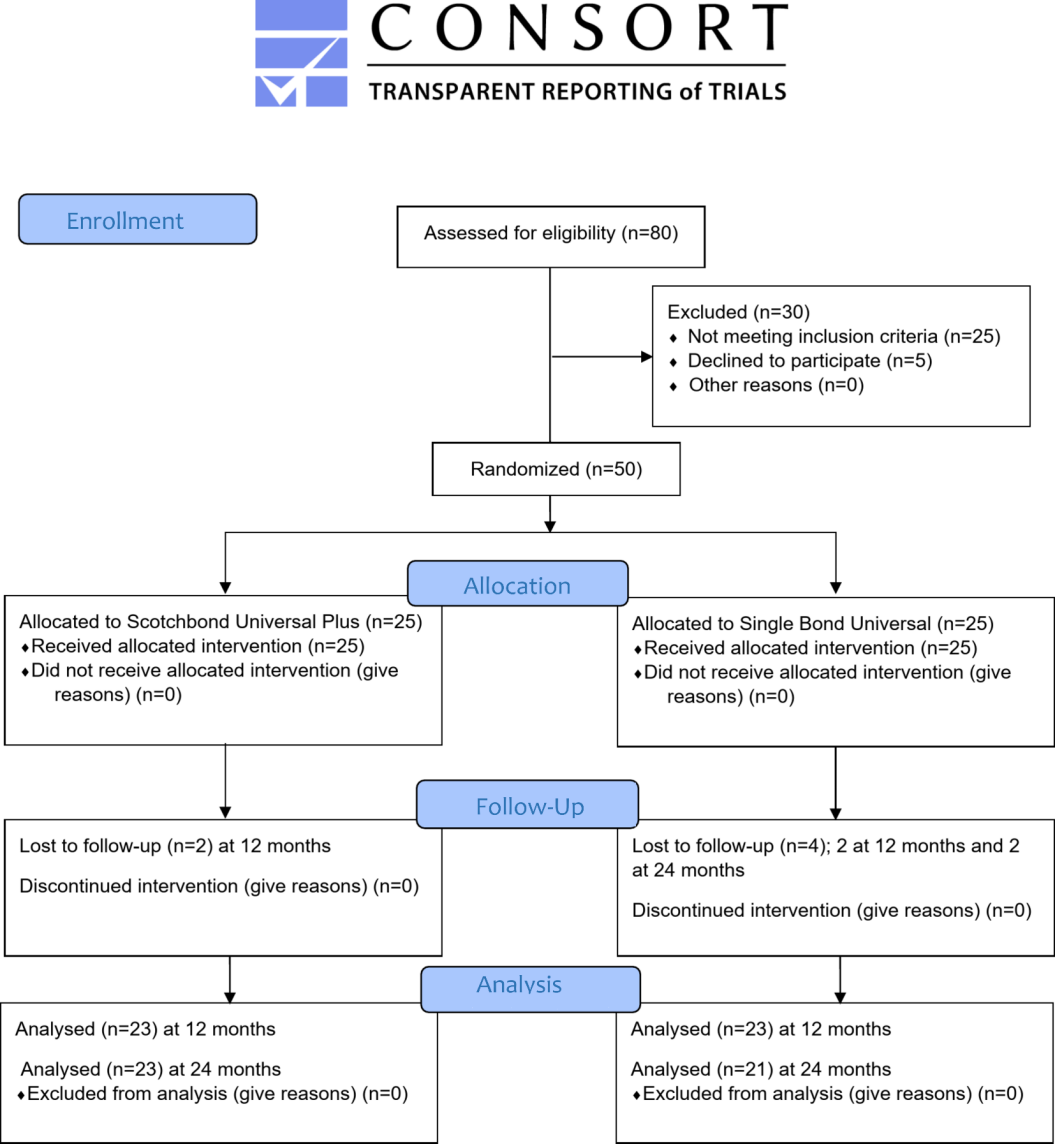
Consort flow diagram.

This present study was conducted on 50 patients with 50 cervical carious lesions. After 24 months 44 restorations were assessed with 88% retention rate, six patients were dropped-out; four at 12 months and two at 24 months follow-up. CONSORT flow diagram illustrates participant flow through each stage of the trial (Fig. 2). The mean age of the participants in the current trial was 28.5 ± 5.7 years, there was no statistically significant difference between both groups regarding age (*p* = 0.511). Additionally, there was 12 males and 38 females in the current study, there was no statistically significant difference between both groups in gender distribution (*p* = 0.5121).

### Clinical evaluation

The comparison between adhesives showed no statistically significant differences within all follow-up periods regarding all tested parameters (*p* > 0.05). Intragroup comparison between follow-up periods within both adhesives has shown no statistically significant change in scores for all tested outcomes (*p* > 0.016)except for marginal adaptation within single bond universal, where there was statistically significant difference (*p* = 0.005). At baseline, two restorations in the Scotchbond™ Universal Plus and four in the Single Bond Universal group exhibited postoperative sensitivity, which resolved in subsequent follow-ups. After 24 months, there was 87% less risk for score B in Scotchbond™ Universal Adhesive Plus when compared to Single Bond Universal (RR = 0.13, 95% CI 0.007161 to 2.3948, *p* = 0.1704) (Table 3).

**Fig. 3:**
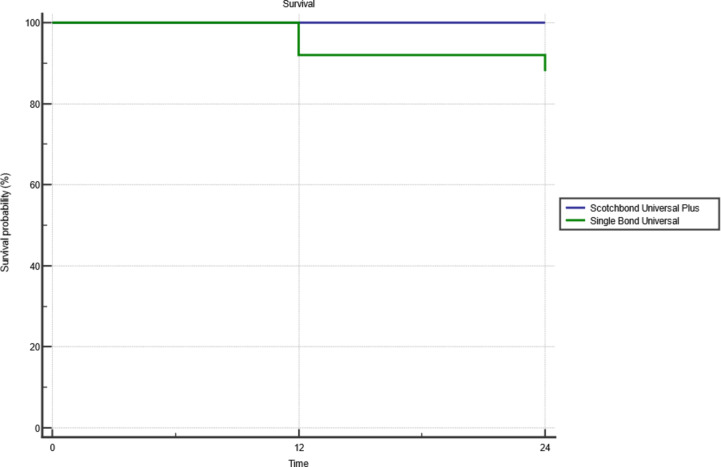
Survival analysis of Scotchbond™ universal adhesive plus and single bond universal for class V restorations after 24 months.

Overall survival of Scotchbond™ Universal Plus and Single Bond Universal for carious cervical restorations was assessed after 24 months, no restorations scored B or C in Scotchbond™ Universal Adhesive Plus group after 24 months. However, in Single Bond Universal group three restorations scored B after 12 and 24 months in marginal adaptation and marginal discoloration. Kaplan-Meier analysis and Log-rank test showed no statistically significant difference between both materials (*p* = 0.0769) (Fig. 3).

**Table 3:**
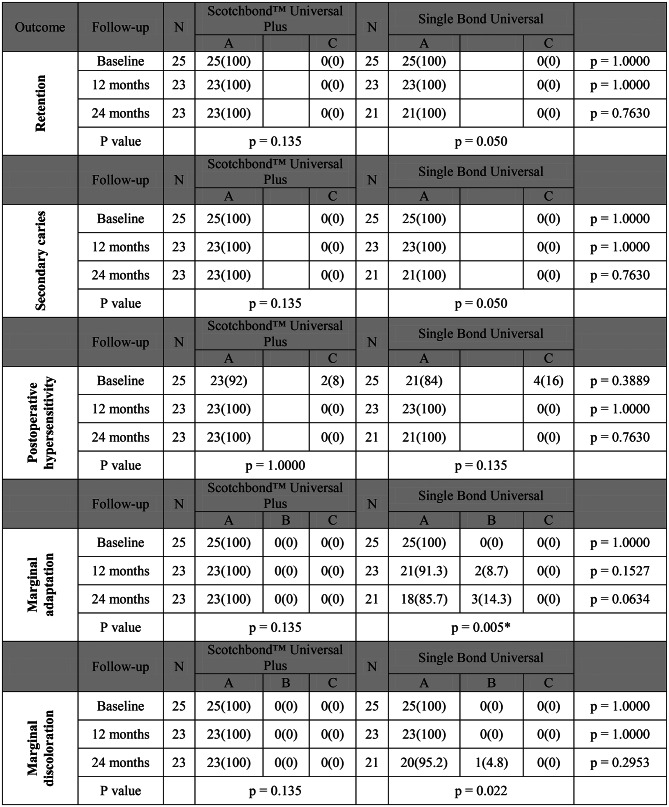
Frequency (n) and percentage (%) of modified USPHS criteria showing retention, secondary caries, post-operative hypersensitivity, marginal adaptation and marginal discoloration. *Denotes statistically significant difference.

## Discussion

After 24 months, cervical restorations in Scotchbond™ Universal Adhesive Plus showed 100% alpha score, while in Single Bond Universal group cervical restorations showed 85.7% alpha score, yet all restorations were clinically successful. There was no statistically significant difference between both adhesives for all tested criteria after 24 months (*p* > 0.05), therefore, the null hypothesis cannot be rejected. The two adhesives showed similar clinical performance with regard to retention, postoperative sensitivity, and secondary caries. This may be due to similar composition of both adhesive mainly the functional monomers (10-MDP), Vitrebond co-polymer and HEMA, in addition to the application protocol and mild pH of 2.7^13,20^.

The MDP monomer enhances adhesion to tooth structure through a chemical bond with hydroxyapatite, a process referred to nano-layering. Additionally, the Vitrebond copolymer facilitates an ionic interaction between the carboxyl groups in polyalkenoic acid and hydroxyapatite in both enamel and dentin. This chemical reaction is considered fundamental to the bonding mechanism. The inclusion of the HEMA monomer makes the adhesive hydrophilic, which improves its wettability on the tooth surface^21^.

Current evidence suggests that for cervical lesions, universal adhesives should ideally be used with an etch-and-rinse protocol, which a previous systematic review concluded that it yields the best clinical outcomes^22^. According to Hong et al.^23^, the etch-and-rinse approach for universal adhesives provides superior clinical benefits, including improved retention, better marginal adaptation, and reduced marginal discoloration compared to the self-etch mode. However, Rodriguez et al. found no significant difference between the two adhesive strategies, indicating that the clinician’s preference and the specific clinical context are key factors in selecting the technique^24^. Therefore, etch and rinse approach for universal adhesives was preferred in the present trial as it provides better retention of cervical restorations up to 36 months than self-etch approach^25^.

The bonding performance of adhesive agents is typically assessed by evaluating clinical performance in cervical restoration^26^. To ensure standardized quality in clinical evaluations, various criteria have been developed, with the United States Public Health Service (USPHS) criteria being among the most widely adopted^27^. Numerous clinical trials utilizing modified USPHS criteria support their validity and reliability^3^.

Regarding retention, no restoration loss occurred in either group during the trial, resulting in a 100% retention rate. This high retention has been similarly reported by previous trials for Single Bond Universal when using the etch-and-rinse technique^16,28,29^. The excellent retention can be attributed to the chemical bonding from the 10-MDP monomer and Vitrebond copolymer, as previously discussed. This finding aligns with Carvalho et al.^26^, who studied the bond durability of a mild two-step self-etch adhesive containing 10-MDP as a functional monomer and observed favorable results. Additionally, Alam et al.^13^ indicated that the survival rates of Scotchbond™ Universal Plus Adhesive and Scotchbond™ Universal Adhesive were comparable, regardless of the etching method, reflecting adequate bond strength and strong adhesive adaptation.

The present study found no statistically significant difference in postoperative sensitivity between the two groups, aligning with previous clinical trials^16,29–31^. At baseline, two cases in the intervention group and four in the control group exhibited postoperative sensitivity, which resolved in subsequent follow-ups. This sensitivity could have been related to factors other than the adhesive material, such as dentin etching, desiccation, gingival retraction that may expose the root surface immediately after restoration placement, finishing, polishing, or operational stress^28,32^.

Concerning secondary caries, neither of the universal adhesives showed any reports of secondary caries and there were no statistically significant differences between the two materials at all follow-up intervals. Previous research has explored the effect of 10-MDP on caries inhibition potential, highlighting the significance of the acid-base resistant zone, which differs from the conventional hybrid layer and fluoride-releasing caries inhibition zone. This resistant zone is thought to play a crucial role in preventing secondary caries by sealing restoration margins and enhancing restoration longevity^33,34^.

Marginal adaptation is a crucial indicator of the durability of dental restorations. Insufficient marginal integrity can lead to various complications, including gap formation, microleakage, recurrent caries, postoperative hypersensitivity, and ultimately pulp involvement^35^. In the present study, Scotchbond™ Universal Plus showed 100% alpha score in marginal adaptation across various follow-up periods. However, Single Bond Universal showed statistically significant deterioration in marginal adaptation with 3 restorations scoring bravo after 24 months. The relatively satisfactory adaptation scores can be attributed to the etching protocol and the robust chemical reaction facilitated by the 10-MDP monomer and the Vitrebond copolymer, as previously suggested in research^13,21,29^. Factors such as the etching protocol and agitation of the adhesive may have also improved bond strength and clinical performance^36^, allowing for effective penetration of resin tags regardless of the adhesion protocol^37^.

The differences in adhesive thickness between Scotchbond™ Universal Plus and Single Bond Universal adhesives may have contributed to their varying behaviours in marginal quality. Alam et al. found a significant difference in viscosity, with Scotchbond™ Universal Plus having a mean viscosity of 50.2 ± 0.3 MPa compared to 115.5 ± 0.6 MPa for Single Bond Universal. The lower viscosity of Scotchbond™ Universal Plus can improve its wettability over the tooth surface, enhancing adaptation. While higher viscosity typically correlates with better mechanical properties, Scotchbond™ Universal Plus exhibited superior mechanical qualities despite its lower viscosity, likely due to its modified composition^13^. Tsujimoto et al. found a positive correlation between the thickness of the adhesive layer and its bond strength. Scotchbond™ Universal Plus had a thinner adhesive layer in etch and rinse mode of 2.9 ± 0.2 μm, when compared to Single Bond Universal which showed an adhesive layer thickness of 6.1 ± 0.4 μm^38^.

Regarding marginal discoloration, no statistically significant differences were observed between the two groups at various intervals. Only one restoration in the control group received a Bravo score at the 24-month follow-up. The main factors to consider for this outcome are the use of the etch-and-rinse mode and the effect of 10-MDP and Vitrebond as previously mentioned. Higher rates of marginal discoloration have been noted in restorations with universal adhesives using the self-etch method, likely due to their reduced effectiveness in bonding to unetched enamel when compared to etched enamel^30^.

Marginal discoloration is often attributed to microleakage, allowing oral fluids and bacteria to penetrate^39^. However, Kim et al. noted that marginal discoloration does not always indicate microleakage; only penetrating discoloration signifies its presence, while superficial discoloration may result from marginal chipping without evidence of microleakage. In such cases, repair or refurbishment could be a more conservative approach than complete restoration replacement^4^.

Few trials are available in the literature regarding restoration of carious cervical lesions and root caries. A previous clinical trial by Abdalla and Garcia-Godoy found 100% retention rate of cervical restorations using resin composite after two years using etch and rinse approach, selective enamel etching and self-etch approach^40^. Another trial by Nassar et al. found 81.5% survival of resin composite restorations preceded by self-etch adhesive after one year^41^. Moreover AlHumaid et al. found 100% retention rate of flowable composite preceded by etch and rinse approach in cervical lesions after 18 months^42^. Vural et al. also found 85% success rate of root caries restorations after five years using resin composite preceded by self-etch adhesive^43^. A second study by Vural et al. found 84.3% retention rate of cervical restorations using resin composite preceded by etch and rinse adhesive system after three years^44^. The previous clinical trials support the finding of the present trial with success rate ranging from 80 to 100% after one to five years of clinical service.

In the present study six patients were dropped out due to not responding to the follow up recalls, four patients were dropped out at 12 months follow-up and two patients were dropped out at 24 months follow-up with 88% retention rate after 24 months, the retention rate was 92% in the intervention group and 84% in the control group. The two restorations lost due to follow-up at 24 months in Single Bond Universal group scored alpha at 12 months follow-up in all assessed criteria, accordingly it was unlikely to deteriorate based on the performance of other restorations in this group. According to literature, there is 25–26% participants drop-out in clinical trials^45^. In the present clinical trial during sample size calculation 15% was added to compensate for possible dropouts, however only 12% dropped out at the end of the trial in both group, which was less than previously mentioned average dropout rate, therefore the power of the sample size was not affected.

To our knowledge, the current study was pioneer in assessing the clinical performance of Scotchbond™ Universal Adhesive Plus with its modified formula. According to ADA standards, for full acceptance of an adhesive restorations, clinical failures such as loss of restorations and microleakage should be limited to 10% after 18 months^46^. There were no failures after 24 months, therefore, both adhesive materials can be recommended for restoration of carious cervical lesions. One of the limitations in the present study is the relatively small sample size. A sufficient sample size is recommended to detect any differences between both test groups with enhanced power and external validity. Moreover, extending the follow-up to at least three years is recommended in order to grant the full acceptance for adhesive materials^47^.

## Conclusions

The newly introduced version of universal adhesive “Scotchbond™ Universal Plus Adhesive” showed satisfactory clinical performance, comparable to its predecessor Single bond Universal Adhesive after 24 months, despite its modified formula. The new formula enhanced the marginal quality of the upgraded version of universal adhesive. A larger sample size is needed to validate the findings of this study, along with extended follow-up periods to identify any long-term failures associated with both adhesives.

## Data Availability

The datasets used and/or analysed during the current study available from the corresponding author on reasonable request.
