# Author Correction: 24-Month clinical evaluation of cervical restorations bonded using radio-opaque universal adhesive compared to conventional universal adhesive in carious cervical lesions: a randomized clinical trial

**DOI:** 10.1038/s41598-025-93290-0

**Published:** 2025-03-24

**Authors:** Basma Dawoud, Eman Abou-Auf, Omar Shaalan

**Affiliations:** Conservative Dentistry Department, Faculty of Dentistry, Cairo University, Al Saraya Str. 11, Manial, Cairo, Egypt

Correction to: *Scientific Reports*, 10.1038/s41598-025-88201-2, published on 14 February 2025.

The original version of this Article contained an error in Table 3, where the N value was incorrect for ‘24 months’ for the Outcome ‘Postoperative hypersensitivity’. The correct and incorrect values appear below.

Incorrect:
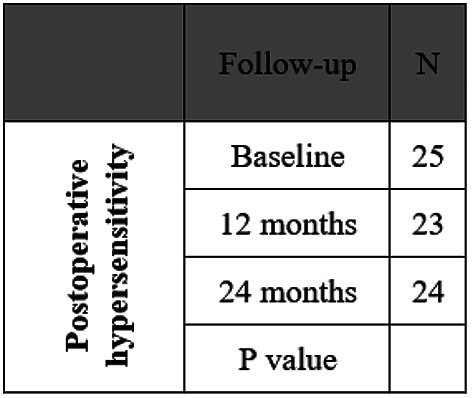


Correct:
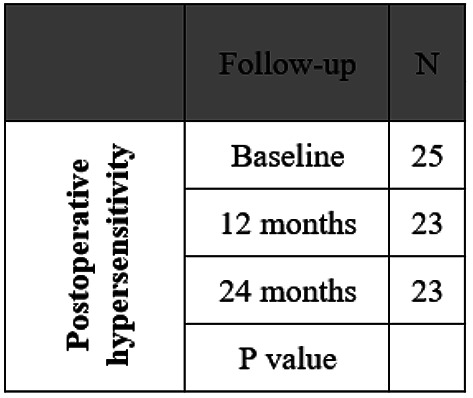


The original article has been corrected.

